# Agreement of WebCeph-Based Automated and Expert-Adjusted Cephalometric Analyses with Manual and Dolphin Tracings

**DOI:** 10.3390/diagnostics16121836

**Published:** 2026-06-13

**Authors:** Güray Gürler, Mustafa Serdar Toroglu, Oruc Yener Cam

**Affiliations:** 1 Department of Orthodontics, Faculty of Dentistry, Cukurova University, 01330 Adana, Turkey; storoglu@cu.edu.tr; 2 Private Practice, 01130 Adana, Turkey; yenercam@gmail.com

**Keywords:** artificial intelligence, cephalometric analysis, expert-adjusted AI, lateral cephalogram, WebCeph

## Abstract

**Background**: This study aimed to compare the measurement agreement and intramethod reliability of four cephalometric analysis workflows: manual tracing, semi-automated digital analysis (Dolphin), fully automated AI-based analysis (WebCeph), and expert-adjusted AI analysis (WebCeph+). **Methods:** In this retrospective method-comparison study, 67 lateral cephalometric radiographs were initially included. After the exclusion of radiographs containing extreme values, 54 radiographs (35 females, 19 males; mean age: 15.0 ± 2.13 years) were analyzed. Twenty-one skeletal, dental, and soft-tissue parameters (13 angular, 8 linear) were evaluated across the four methods. Intramethod repeatability was assessed via the intraclass correlation coefficient (ICC). Intermethod comparisons were analyzed using ANOVA and post hoc pairwise tests. Pragmatic clinical relevance thresholds were predefined as ±2 degrees for angular measurements and ±2 mm for linear measurements. **Results:** All methods demonstrated high intramethod reliability, with ICC values exceeding 0.90 in 20 out of 21 parameters. Manual and Dolphin methods yielded statistically comparable results (*p* > 0.05). In contrast, WebCeph differed significantly from manual and/or Dolphin in seven parameters, including SNA, IMPA, Go-Gn length, Pog to N-perpendicular, Wits appraisal, nasolabial angle, and mentolabial angle (*p* < 0.05). Several discrepancies exceeded the predefined pragmatic thresholds (±2 degrees and ±2 mm), highlighting their potential clinical relevance. After expert adjustment (WebCeph+), statistically significant inter-workflow differences were no longer observed; however, residual individual-level variability remained for selected parameters. **Conclusions:** Fully automated WebCeph analysis showed limited agreement with manual and semi-automated methods for several clinically relevant measurements. Expert adjustment reduced systematic mean discrepancies and improved agreement with clinician-dependent workflows; however, residual individual-level variability remained for selected parameters. AI-driven cephalometric analysis should therefore be considered a supportive tool requiring specialist verification rather than an unsupervised replacement for conventional methods.

## 1. Introduction

Cephalometric analysis is essential for diagnosing malocclusion, evaluating dentofacial and soft-tissue structures, and monitoring growth- or treatment-related changes [1,2,3]. Since its introduction by Broadbent, it has remained indispensable in orthodontic diagnosis and treatment planning [4,5].

Conventional cephalometric analysis involves the manual identification of anatomical landmarks on acetate tracings, followed by linear and angular measurements [6,7,8]. Although widely accepted, this workflow is time-consuming and dependent on the clinician’s experience, while landmark identification remains prone to random error [9,10].

With advancements in technology, cephalometric analyses have transitioned to digital platforms, partially overcoming these limitations [11,12]. Although anatomical landmarks are still manually identified, computer-assisted cephalometric analysis software has improved time efficiency and reduced measurement errors [13,14,15]. Additionally, the adoption of digital workflows, including Digital Imaging and Communications in Medicine (DICOM)-compliant image acquisition, picture archiving and communication system (PACS)-based storage and retrieval, and standardized sharing protocols, has facilitated image capture and archiving, improved interoperability among clinicians and systems, and shortened the time required for superimposition [16,17]. Despite these advantages, the primary source of error in computerized cephalometric systems continues to be the manual identification of anatomical landmarks, which remains operator-dependent and prone to intra- and inter-examiner variability [18,19].

Artificial intelligence, particularly deep learning, has been increasingly adopted in medicine and dentistry, leading to the development of fully automated AI-assisted cephalometric analysis software [20,21]. In orthodontics, such fully automated systems aim to minimize errors in case analysis [22,23] and improve clinical efficiency [23,24,25]. WebCeph, a South Korea–based platform, is a web-based, fully automated cephalometric analysis system driven by artificial intelligence. It supports essential orthodontic functions, including patient record management, cephalometric analyses, growth monitoring, treatment simulation, and treatment planning [26].

Despite these potential advantages, the accuracy and clinical interchangeability of fully automated cephalometric analysis remain uncertain. A recent diagnostic comparison by Sadek et al. [27] showed that fully automated systems produced statistically significant deviations from reference tracings in both skeletal and soft-tissue parameters, despite their speed advantages. Similarly, evidence from automated landmark detection studies and a recent systematic review and meta-analysis indicates that mean radial landmark errors remain close to the 2-mm threshold, with success detection rates within 2 mm ranging from 58% to 72%, suggesting that clinically relevant discrepancies may persist in automated workflows [28,29,30]. In an earlier systematic review, Leonardi et al. [31] reported that automatic cephalometric analysis systems exhibited greater landmark-identification errors than manual tracing, highlighting longstanding concerns regarding their agreement with conventional methods. Conversely, some studies argue that artificial intelligence achieves accuracy comparable to manual tracing [26,32].

Recent studies have specifically examined AI-assisted cephalometric workflows in relation to semi-automated and corrected landmark outputs. Raby et al. compared WebCeph and CephX in both automated and landmark-corrected forms with Dolphin Imaging and reported that landmark correction improved the agreement of AI-generated measurements with the semi-automated workflow [33]. In addition, Zughair et al. compared AI-based automatic, semi-automatic, and manual digital cephalometric tracing methods and reported method-dependent differences in selected skeletal and soft-tissue measurements [34].

Nevertheless, even advanced AI-driven platforms still exhibit higher random errors and small but systematic biases compared with expert manual tracings, limiting their full interchangeability in orthodontic decision-making [35,36]. Although recent evidence has demonstrated the potential benefit of correcting AI-generated landmarks, further evaluation is warranted to determine the extent to which expert adjustment of WebCeph output improves agreement with both conventional manual tracing and semi-automated digital analysis across skeletal, dental, and soft-tissue parameters under standardized imaging conditions.

The aim of this study was to compare cephalometric analysis results obtained using WebCeph, an AI-based fully automated cephalometric analysis platform, with those from a computer-assisted semi-automated program (Dolphin) and conventional manual tracing, and to determine whether expert adjustment of AI-generated landmarks (WebCeph+) can improve agreement with clinician-dependent methods. Additionally, the study sought to evaluate the intramethod repeatability of all four workflows. The null hypothesis (H_0_) was that there would be no significant difference among manual tracing, computer-assisted semi-automated analysis, fully automated AI-based analysis, and expert-adjusted AI analysis in angular and linear measurements obtained from lateral cephalometric radiographs.

## 2. Materials and Methods

Ethical approval for this retrospective study was obtained from the Institutional Research Ethics Committee (Meeting No.: 154; Decision No.: 27; Date: 18 April 2025). The records, clinical data, and radiographic images of patients who presented for orthodontic treatment between 2018 and 2023 were reviewed. This study was conducted in accordance with the Declaration of Helsinki (version 2008). Written informed consent for the use of anonymized radiographic records for research purposes had been obtained at the time of routine clinical admission, in accordance with institutional policy.

### 2.1. Study Design

This retrospective, observational and analytical method-comparison study evaluated the measurement agreement and intramethod repeatability of cephalometric measurements obtained using manual, semi-automated, and fully automated analysis methods. Data obtained from fully automated analyses following expert adjustment were analyzed as a separate group.

### 2.2. Sample Size Calculation

The sample size was calculated using power analysis (G*Power, version 3.1.9.2, Franz Faul; University of Kiel, Kiel, Germany) based on a previous study [37] with a power of 90%, an α error of 5%, and an effect size of 0.5. The minimum required sample size was 54 lateral cephalometric radiographs. To account for possible exclusions related to statistically extreme values during the planned data-screening procedure, the initial sample size was increased to 67 radiographs.

### 2.3. Eligibility Criteria

Digital lateral cephalometric radiographs acquired using the same device were included, provided that they were obtained from patients aged 12 years or older with complete permanent dentition and no prior orthodontic treatment. The lower age threshold of 12 years was selected to ensure inclusion of patients in the permanent dentition stage and to reduce variability related to mixed dentition, transitional eruption status, superimposition of developing or erupting structures, and age-dependent differences in landmark interpretation. Only radiographs obtained with the teeth in maximum intercuspation and free from metal structures that could interfere with the identification of anatomical landmarks were considered. Radiographs were included only if bilateral anatomical landmarks exhibited acceptable superimposition on the midsagittal plane and image quality was sufficient for accurate landmark identification.

Radiographs were excluded if they were obtained from patients with craniofacial anomalies, exhibited impacted or missing teeth (excluding third molars), or contained pathological findings or artefacts. Additionally, images in which anatomical reference points could not be clearly identified due to motion blur, insufficient resolution, or poor contrast were also excluded.

In accordance with these criteria, digital radiographs of 192 patients were initially reviewed. A total of 125 radiographs were excluded because they did not meet the eligibility criteria, resulting in 67 radiographs eligible for the initial methodological evaluation. Statistical handling of extreme values was performed subsequently and independently of the eligibility assessment, as described in the Section 2.7. No distinction was made between different types of malocclusion.

### 2.4. Cephalometric Imaging Protocol

All lateral cephalometric radiographs were obtained using the Planmeca Promax Dimax4Ceph device (Planmeca Oy, Helsinki, Finland) with exposure parameters set at 75 kV, 78 mA, and an exposure time of 1.87 s. The radiographs were digitally stored in JPEG format. During image acquisition, patients were positioned with the Frankfort horizontal plane (FH) parallel to the floor; the teeth were in maximum intercuspation, and the lips were in a relaxed position.

### 2.5. Cephalometric Analysis Methods

All human-dependent tracings were performed by a single examiner with 10 years of clinical experience.

#### 2.5.1. Calibration Protocol

For all methods, calibration was performed to ensure 1:1 (pixel-to-millimeter) scaling. A manufacturer-provided scale marker/ruler visible on the source images was used in all workflows to preserve magnification and measurement comparability across methods. In manual tracing, calibration was based on a 40 mm segment of this ruler, whereas in the digital methods (Dolphin and WebCeph), the on-image scale marker was registered to ensure 1:1 pixel-to-millimeter output. All analyses used identical source images with preserved native resolution.

#### 2.5.2. Manual Tracing Method

Digital radiographs in JPEG format were imported into Adobe Photoshop CC 2020 (v21.2.12, Adobe Systems Inc., San Jose, CA, USA), calibrated to a 1:1 ratio, and printed on A3-sized paper. Each radiograph was manually traced by the same investigator using a 0.35 mm mechanical pencil, a digital protractor, and a ruler. A3-sized prints were used to preserve image size after 1:1 calibration and to facilitate visualization of bilateral structures and soft-tissue contours under standardized lighting conditions.

#### 2.5.3. Semi-Automated Digital Method (Dolphin)

Dolphin Imaging (v11.95; Dolphin Imaging and Management Solutions, Chatsworth, CA, USA) was used for semi-automated digital analysis. In this workflow, the examiner manually identified the cephalometric landmarks using a computer mouse, after which the software automatically calculated the selected angular and linear measurements. No artificial intelligence-based or automated landmark detection function was used in the Dolphin analyses performed in this study.

#### 2.5.4. Fully Automated Digital Method (WebCeph)

WebCeph (AssembleCircle Co., Ltd., Seoul, Republic of Korea) was used as a web-based, artificial intelligence-assisted, fully automated cephalometric analysis platform. Anatomical landmarks were automatically identified through the “AI Digitization” function, and all measurements were generated automatically. WebCeph has been described as using a deep learning-based approach for automated cephalometric landmark detection and analysis [26]. However, the detailed architecture, training dataset, and version-specific algorithmic characteristics of the commercial platform used in the present study are not publicly disclosed; therefore, no further technical description of the proprietary algorithm can be provided.

#### 2.5.5. Analysis Following Investigator Intervention (WebCeph+)

The anatomical landmarks automatically placed by WebCeph were individually reviewed by the investigator. Landmarks were manually repositioned only when the automatically assigned point was judged to be clearly misplaced relative to the intended anatomical landmark on visual inspection of the source image. If the automatically identified position was considered acceptable, no manual correction was performed. Each workflow was completed separately, and the examiner did not alternate between methods within the same case during landmark identification. The calibrated inputs and 1:1 scaling established in WebCeph were preserved without any resampling or image manipulation. The results obtained after this intervention were categorized into a separate analysis group labeled “WebCeph+”. During the WebCeph+ correction procedure, the investigator was not blinded to the manual and Dolphin measurements, as the primary objective was to assess whether expert intervention could improve the agreement of AI-generated measurements with the clinician-dependent workflows.

**Figure 1:**
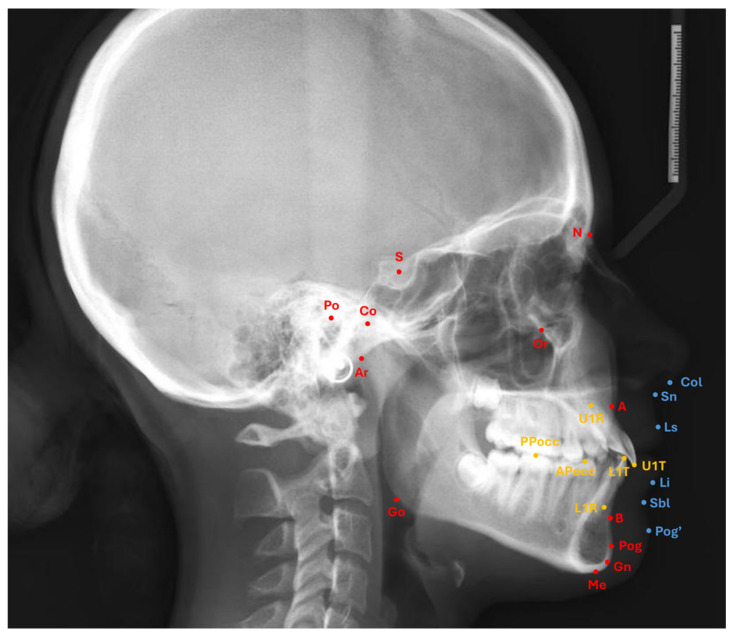
Lateral cephalogram with cephalometric landmarks employed for angular and linear measurements. Red points indicate skeletal structures, including Sella (S), Nasion (N), Porion (Po), Orbitale (Or), Condylion (Co), Articulare (Ar), Point A, Point B, Pogonion (Pog), Gnathion (Gn), Menton (Me), and Gonion (Go). Yellow points represent dental landmarks such as the tip and root apex of the upper central incisor (U1T and U1R), the tip and root apex of the lower central incisor (L1T and L1R), and occlusal plane reference points including the anterior and posterior occlusal contacts (APocc and PPocc). Blue points denote soft-tissue profile markers, comprising Columella (Col), Subnasale (Sn), Labrale Superius (Ls), Labrale Inferius (Li), Sublabiale (Sbl), and soft tissue Pogonion (Pog’). Scale bar = 45 mm.

**Figure 2:**
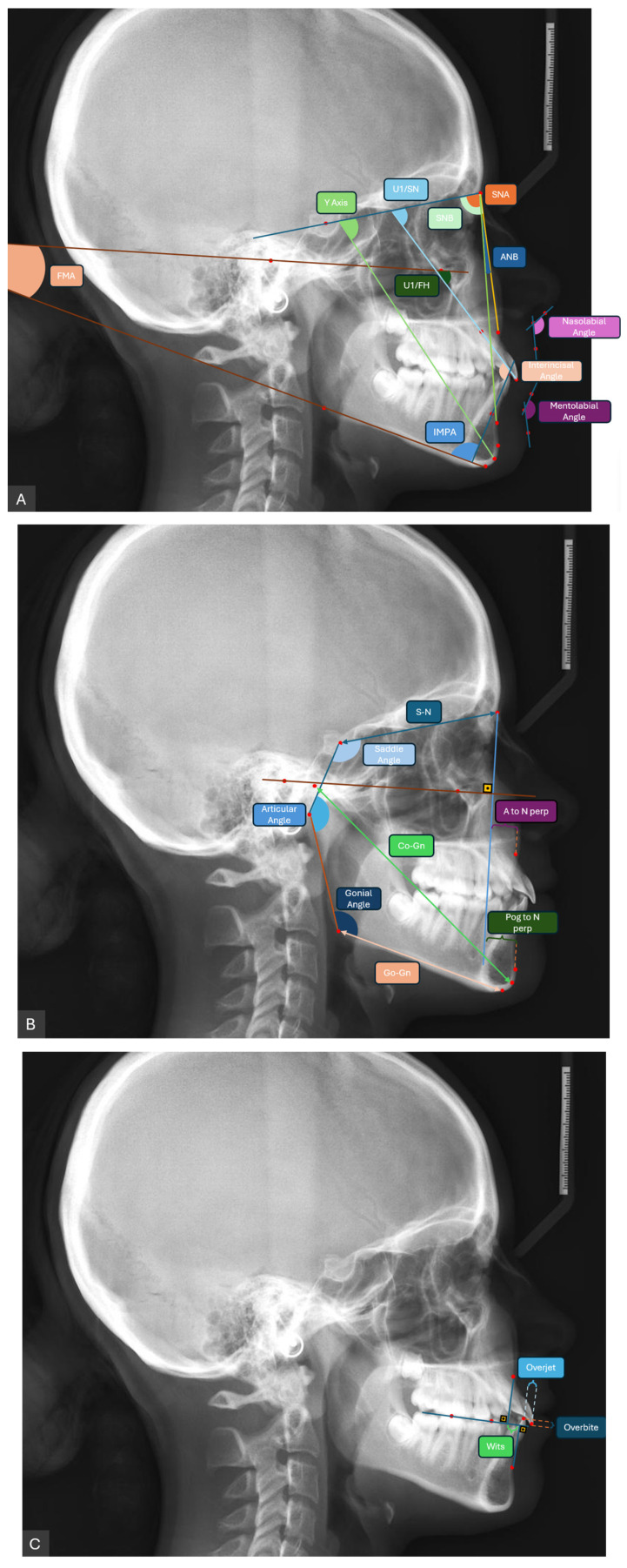
Cephalometric angular and linear measurements illustrated on lateral cephalometric radiographs. Point A and Point B refer to the anatomical landmarks defined in Figure 1. (**A**) Angular measurements: SNA (Sella-Nasion-A point), SNB (Sella-Nasion-B point), ANB (A point-Nasion-B point), FMA (Frankfort-mandibular plane angle), Y-axis (Sella-Gnathion), U1/SN (upper central incisor to Sella-Nasion), U1/FH (upper central incisor to Frankfort horizontal), IMPA (lower incisor to mandibular plane), Interincisal angle, Nasolabial angle, Mentolabial angle. (**B**) Angular measurements: Saddle angle (Nasion-Sella-Articulare), Articular angle (Sella-Articulare-Gonion), Gonial angle (Articulare-Gonion-Menton). Linear measurements: S-N (Sella-Nasion), Co-Gn (Condylion-Gnathion), Go-Gn (Gonion-Gnathion), A to N-perpendicular (A point to Nasion perpendicular), Pog to N-perpendicular (Pogonion to Nasion perpendicular). (**C**) Linear measurements: Overjet, Overbite, Wits appraisal (projection of points A and B on the occlusal plane). Colored lines indicate cephalometric planes and linear measurements; red dots indicate anatomical landmarks; arcs indicate angular measurements; dashed lines indicate perpendicular or projected linear measurements; and square symbols indicate right angles. Scale bar = 45 mm.

Across all methods, a total of 21 cephalometric parameters were measured, including 13 angular and 8 linear variables, based on 24 anatomical landmarks (Table 1) (Figure 1 and Figure 2). The selected 24 landmarks and 21 measurements were chosen to provide a balanced assessment of skeletal, dental, and soft-tissue relationships and to include parameters that are routinely used in orthodontic diagnosis and repeatedly evaluated in previous method-comparison studies. Priority was given to commonly reported variables that represent sagittal and vertical skeletal relationships, incisor inclination, mandibular length, sagittal jaw discrepancy, and profile morphology.

### 2.6. Assessment of Method Error

Repeatability was assessed on a randomly selected subset of eight radiographs (14.8% of the final primary analytic sample) across all four workflows. The subset size was determined during the initial statistical planning stage using the approach $n=1+\sqrt{N}$, where N  represented the final primary analytic sample size. Based on the final primary analytic sample size of 54 radiographs, this approach yielded a repeatability subset of eight radiographs. The radiographs were randomly selected from an alphabetically ordered participant list using Microsoft Excel Professional Plus 2019 (Microsoft Corp., Redmond, WA, USA). In the human-dependent workflows (manual tracing, Dolphin, and WebCeph+), the same examiner repeated landmark identification and measurement procedures after a wash-out interval without access to the previous measurements of the corresponding workflow. In the fully automated WebCeph workflow, the same image set was reprocessed to assess deterministic output reproducibility. All 21 cephalometric parameters were re-measured in this subset, and intramethod intraclass correlation coefficients based on a two-way mixed-effects model for absolute agreement, together with 95% confidence intervals, were calculated.

### 2.7. Statistical Analysis

SPSS 26.0 software was used for statistical analysis (IBM Corp., Armonk, NY, USA). The normality of age distribution was assessed using the Kolmogorov–Smirnov test, and the Mann–Whitney U test was applied for age comparisons between groups. Categorical demographic variables were compared using the chi-square test. The distribution of quantitative variables was assessed using the Kolmogorov–Smirnov test because the sample size exceeded the conventional threshold for the Shapiro–Wilk test (*n* ≤ 50), above which the two tests show comparable power. In addition, Q–Q plots and histograms were visually inspected to verify distributional assumptions.

For each of the 21 evaluated cephalometric parameters, Z scores were calculated using the formula $(x_{i}-\mu )/\sigma$. Values with an absolute Z score greater than 3 were considered statistically extreme values. A radiograph-level exclusion rule was applied in the primary analysis: any radiograph containing at least one statistically defined extreme value across the 21 evaluated parameters was excluded from the primary analytic dataset. This radiograph-level exclusion rule was adopted to avoid selective deletion of individual measurements within the same subject and to preserve within-subject consistency across all four workflow comparisons.

To compare mean parameter values among the four analysis methods, one-way analysis of variance (ANOVA) was used. Each method was treated as a distinct analytical workflow because preprocessing, calibration, and landmarking logic differed across systems. Repeated-measures ANOVA was additionally performed as a sensitivity analysis and yielded the same overall significance pattern, supporting the consistency of the findings. This approach is consistent with previous comparison studies of manual, semi-automated, and AI-assisted cephalometric systems [37,38,39]. Prior to ANOVA, Bartlett’s test was used to assess homogeneity of variance. For parameters with homogeneous variances, the F-test (ANOVA) was applied, whereas Welch ANOVA was used for those with heterogeneous variances. For post hoc pairwise comparisons, Tukey’s test was used for parameters with homogeneous variances, whereas the Games–Howell test was used for those with heterogeneous variances. Statistical significance was set at *p* < 0.05 for all tests. Pragmatic clinical relevance thresholds of ±2° for angular measurements and ±2 mm for linear measurements were adopted in accordance with commonly used criteria in comparative cephalometric studies; however, these limits should be interpreted as pragmatic rather than absolute, as clinically relevant thresholds may vary across studies and clinical contexts [40,41]. Given the exploratory nature of the study, findings close to these thresholds were interpreted cautiously.

Additionally, Bland–Altman analyses were performed for selected clinically relevant parameters showing prominent method-dependent discrepancies in the primary analysis. Agreement was evaluated for manual tracing versus fully automated WebCeph and for manual tracing versus expert-adjusted WebCeph (WebCeph+) by calculating the mean difference (bias) and the 95% limits of agreement, defined as the mean difference ± 1.96 standard deviations of the differences. Differences were calculated as WebCeph/WebCeph+ minus manual tracing.

## 3. Results

**Figure 3:**
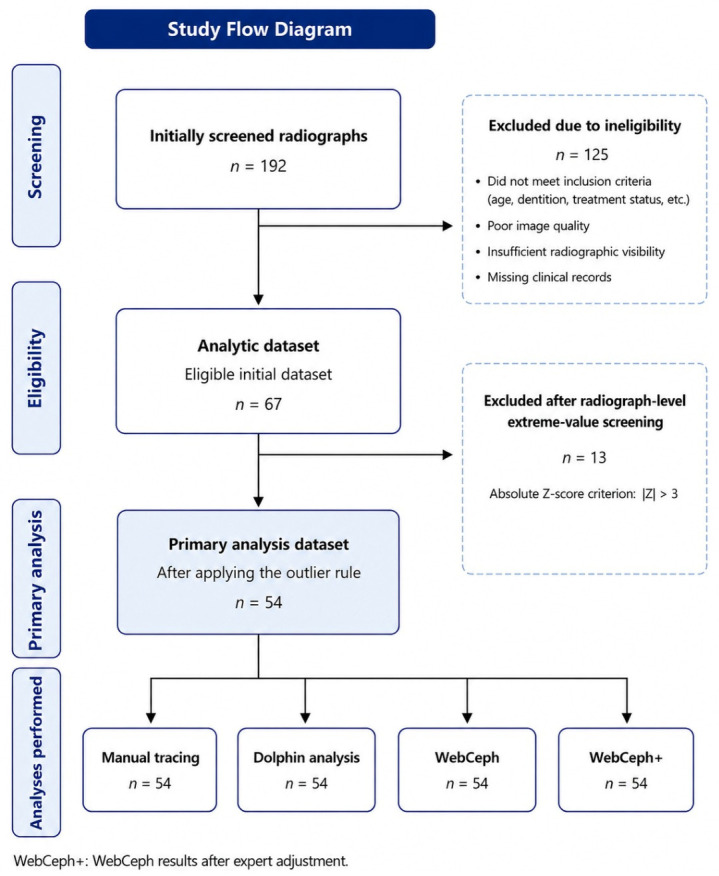
Study flow diagram illustrating the screening of lateral cephalometric radiographs, eligibility-based exclusions, formation of the initial dataset, exclusion after radiograph-level extreme-value screening, and the final primary analytic sample evaluated using manual tracing, Dolphin analysis, WebCeph, and WebCeph+.

A total of 192 lateral cephalometric radiographs were initially screened for eligibility. Of these, 125 radiographs were excluded because they did not meet the predefined eligibility criteria, leaving 67 radiographs in the initial dataset. A total of 21 cephalometric parameters were assessed on these radiographs (Table 1). Outlier screening based on the absolute Z-score criterion of >3 identified 27 extreme values across 15 parameters in 13 radiographs. In accordance with the radiograph-level exclusion rule, these 13 radiographs were excluded from the primary analytic dataset, resulting in a final primary analysis sample of 54 radiographs (Figure 3). The study population consisted of 35 females (64.8%) and 19 males (35.2%), aged between 13 and 23 years. No distinction was made according to malocclusion classification (Table 2).

The overall mean age was 15.0 ± 2.13 years. The mean age of the females was 15.2 ± 2.00 years, whereas that of the males was 14.68 ± 2.33 years. No statistically significant age difference between sexes was found (Mann–Whitney U, *p* > 0.05). The sample was sex-imbalanced, with a higher proportion of females (*p* < 0.05).

The repeatability of measurements was assessed using intraclass correlation coefficients (ICCs). ICC estimates exceeded 0.90 for 20 out of the 21 evaluated parameters, indicating high overall intramethod repeatability across the four workflows. The only parameter with an ICC value below 0.90 was Pogonion to N-perpendicular in the Dolphin workflow (ICC = 0.871). Fully automated WebCeph yielded ICC values of 1.000 for all parameters, reflecting identical outputs when the same images were reprocessed. Following expert adjustment, WebCeph+ maintained high intramethod repeatability across all evaluated parameters (Table 3).

When ICC values were compared across groups, both manual measurements and Dolphin software showed high reliability, with most ICC values above 0.90. In contrast, the fully automated WebCeph analysis produced ICC values of 1.000 for all parameters, reflecting perfect internal consistency. However, after expert adjustment in the WebCeph analysis, minor decreases were observed in certain angular and linear parameters (Table 3).

Pairwise post hoc comparisons indicated no significant differences between manual and Dolphin for any parameters (*p* > 0.05). Relative to manual tracing, WebCeph differed for Go-Gn, Pogonion to N-perpendicular, Wits appraisal, nasolabial angle, and mentolabial angle (*p* < 0.05). Relative to Dolphin, WebCeph differed for SNA, IMPA, Go-Gn, Pogonion to N-perpendicular, Wits appraisal, nasolabial angle, and mentolabial angle (*p* < 0.05) (Table 4). Among angular measurements, the largest discrepancy was observed in the mentolabial angle (8.6° in manual vs. WebCeph and 6.9° in Dolphin vs. WebCeph), while the greatest linear difference was found in the Go-Gn length (2.8 mm and 3.1 mm, respectively).

Following expert adjustment in the WebCeph+ analysis, previously observed differences across all affected parameters were no longer statistically significant. Measurements obtained from WebCeph+ did not differ significantly from either manual or Dolphin analyses (*p* > 0.05) (Table 4). When absolute mean inter-workflow differences were interpreted using the predefined pragmatic clinical relevance thresholds of ±2° for angular measurements and ±2 mm for linear measurements, absolute mean differences involving fully automated WebCeph exceeded these thresholds in 8 of the 21 evaluated parameters when compared with manual tracing and in 9 of the 21 parameters when compared with Dolphin. Following expert adjustment, the number of threshold-exceeding parameters decreased to 1 in the WebCeph+ versus manual comparison and to 2 in the WebCeph+ versus Dolphin comparison. Thus, expert adjustment markedly reduced, but did not entirely eliminate, mean discrepancies exceeding the predefined pragmatic thresholds.

**Figure 4:**
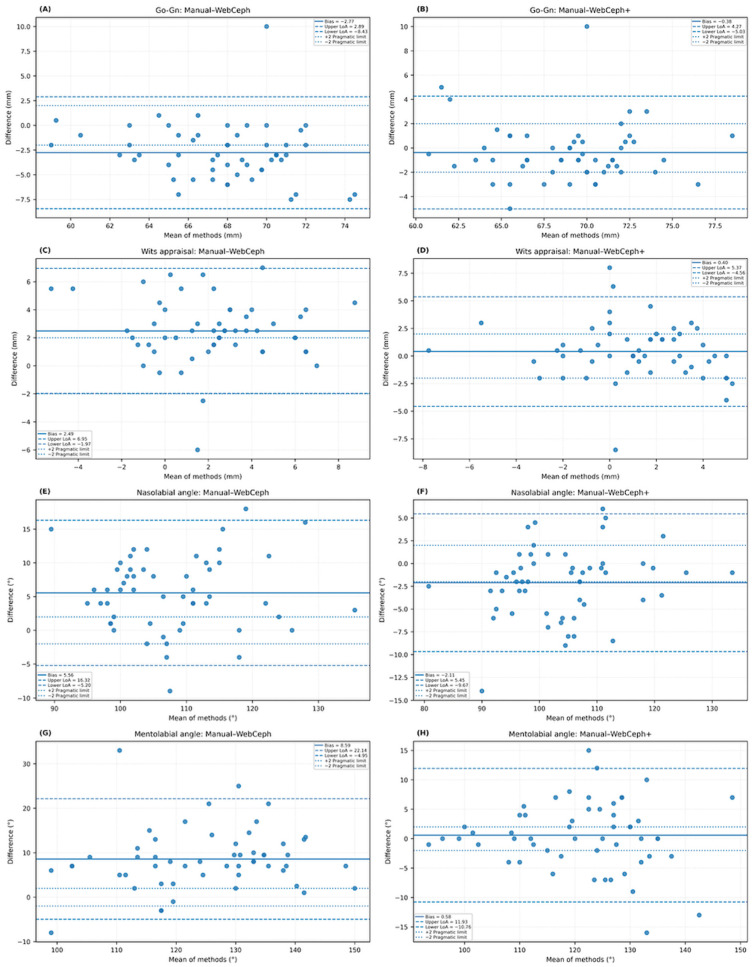
Bland–Altman plots illustrating agreement between manual tracing and fully automated WebCeph, and between manual tracing and expert-adjusted WebCeph (WebCeph+), for selected clinically relevant cephalometric parameters: Go-Gn length (**A**,**B**), Wits appraisal (**C**,**D**), nasolabial angle (**E**,**F**), and mentolabial angle (**G**,**H**). Panels (**A**,**C**,**E**,**G**) represent the comparisons between manual tracing and WebCeph, whereas panels (**B**,**D**,**F**,**H**) represent the comparisons between manual tracing and WebCeph+. Differences were calculated as WebCeph/WebCeph+ minus manual tracing; therefore, negative bias values indicate lower mean measurements for WebCeph/WebCeph+ than for manual tracing. The solid horizontal line represents the mean difference (bias), the dashed horizontal lines represent the upper and lower 95% limits of agreement, and the dotted horizontal lines represent the predefined pragmatic thresholds of ±2 mm for linear measurements and ±2° for angular measurements.

Bland–Altman plots for selected clinically relevant parameters are presented in Figure 4. The absolute mean difference between WebCeph and manual tracing decreased after expert adjustment from 2.77 mm to 0.38 mm for Go-Gn length and from 2.49 mm to 0.40 mm for Wits appraisal. Among the soft-tissue measurements, the absolute mean difference decreased from 5.56° to 2.11° for the nasolabial angle and from 8.59° to 0.58° for the mentolabial angle. These findings indicate that expert adjustment reduced systematic mean discrepancies for the selected parameters. However, the limits of agreement remained relatively wide for several measurements, particularly the soft-tissue parameters, indicating residual individual-level variability despite the reduction in systematic bias.

## 4. Discussion

In this study, manual tracing, a semi-automated system (Dolphin), an AI-powered fully automated system (WebCeph) and expert-adjusted AI analysis (WebCeph+) were compared for measurement agreement and intramethod repeatability using lateral cephalometric radiographs. The main finding was that WebCeph produced statistically significant and pragmatically relevant mean discrepancies in selected skeletal and soft-tissue parameters, whereas expert adjustment (WebCeph+) reduced systematic mean discrepancies and eliminated statistically significant inter-workflow differences. However, the Bland–Altman findings indicated that residual individual-level variability remained for selected parameters. Significant inter-method differences were observed in seven of the twenty-one parameters; therefore, the null hypothesis was rejected for those variables, indicating that the four workflows cannot be considered fully interchangeable across all measurements.

The sample size and parameter selection (21 measurements: 13 angular and 8 linear) were consistent with previous comparison studies [37,39,42], which typically evaluated 9–35 variables, confirming that the present design reflects standard cephalometric research practice. To minimize interobserver variability frequently reported in the literature [42,43], all measurements were performed by a single experienced investigator, ensuring standardization throughout the process. Repeatability was assessed on a randomly selected subset of eight radiographs (14.8% of the sample) across all four workflows using intra-method intraclass correlation coefficients (two-way mixed-effects model, absolute agreement) with 95% confidence intervals. In the human-dependent workflows, this reflected repeated landmark identification by the same blinded examiner, whereas in WebCeph it reflected deterministic output reproducibility on repeated processing of the same image set.

WebCeph yielded ICC values of 1.000 for all parameters. This reflects deterministic algorithmic output rather than superior diagnostic performance, because the software produces identical landmark coordinates for identical inputs. Therefore, a perfect ICC indicates internal computational repeatability rather than the absence of systematic bias. In contrast, the human-dependent workflows (manual, Dolphin, and WebCeph+) naturally exhibited minor variability, which was reflected in slightly lower ICC values despite generally high repeatability. Our main findings revealed that, compared with manual tracing, fully automated analysis by WebCeph showed significant differences in five parameters (Go-Gn length, Pog to N-perpendicular, Wits appraisal, nasolabial angle, and mentolabial angle), and in two additional parameters (SNA and IMPA) when compared with Dolphin. These discrepancies indicate systematic landmarking and reference plane inconsistencies that selectively influence skeletal (sagittal jaw relationships) and soft tissue measurements.

Similar software-dependent offsets in SNA, IMPA and profile-angle measurements were also reported by Bor et al. [13], supporting the reproducibility of these deviations across platforms.

In the assessment of the SNA angle, WebCeph reported values on average 1° higher than manual tracing and 2.21° higher than Dolphin. This discrepancy is likely due to the anterior sagittal positioning of Point A by the AI algorithm. Similar findings have been reported in previous studies, attributing such differences to algorithmic variability in identification of Points A and Nasion [44,45,46]. Point A is particularly challenging to locate accurately due to maxillary alveolar concavities and anatomical superimpositions [45,47]. Furthermore, misidentification of the Nasion point may distort the S-N reference plane, thereby introducing measurement bias in the SNA angle [48]. These observations are supported by reports in the literature indicating lower ICC values for the SNA parameter [42,46]. In a study by Çoban et al. [38], significant differences in SNA measurements between Dolphin and WebCeph were found across different malocclusion types, and these differences were attributed to algorithmic inconsistencies in identifying Point A.

In the Go-Gn parameter, WebCeph produced values, on average, 2.8 mm lower than manual measurements and 3.1 mm lower than Dolphin. These findings suggest that the AI system tends to systematically underestimate mandibular corpus length. This pattern is consistent with documented challenges associated with identifying gonion and gnathion landmarks [39,45,47]. In particular, the gonion point is frequently misidentified because of its anatomically complex structure and bilateral superimposition [48]. Similar inconsistencies have been reported for gnathion localization, highlighting the subjectivity of these landmarks. Consequently, linear measurements such as Go-Gn tend to yield lower ICC values [37].

In the Wits appraisal, WebCeph produced values 2.5 mm higher than manual measurements and 2.3 mm higher than Dolphin. This discrepancy may be attributed to inaccuracies in the perpendicular lines projected from Points A and B onto the occlusal plane [39,47,49]. The anterior displacement of Point A observed in the SNA angle could also have contributed to the deviation in the Wits measurements [45]. Although the literature generally reports high ICC values for this parameter, differences of this magnitude may still hold clinical significance [37,45,46].

For the Pog to N-perpendicular parameter, WebCeph reported values 2.3 mm lower than manual tracing and 2.5 mm lower than Dolphin, indicating a more posterior mandibular position in the sagittal plane. This has been associated with inconsistencies in identification of the pogonion point as well as potential errors in the construction of the N perpendicular plane [37,39,45].

For the dental parameter IMPA, WebCeph reported values that were, on average, 3.87° higher than those obtained using the Dolphin system. This discrepancy has been attributed to the AI algorithm consistently identifying the apical and incisal points of the lower incisors in a more protrusive position [38,39,50]. The literature emphasizes that such differences may be of clinical importance, particularly in influencing treatment decisions such as extraction indications.

Similar divergence between web-based automation and Dolphin has also been documented, particularly for SNA, IMPA and soft-tissue angles [13].

In soft-tissue analysis, differences of 5.6–6° in the nasolabial angle and 6.9–8.6° in the mentolabial angle were observed, indicating that AI algorithms exhibited their weakest performance in identifying soft-tissue landmarks [25,39,43,45]. When interpreted against pragmatic clinical thresholds of ±2° and ±2 mm, these discrepancies clearly exceeded the angular limit and are likely to be clinically perceptible. These thresholds should not be regarded as universally fixed. Although limits of approximately 2° and 2 mm are commonly used to judge the clinical relevance of cephalometric discrepancies, the literature indicates that such thresholds are largely empirical and may vary according to the parameter assessed, the study design, and the clinical context [40,41].

Notably, statistically significant inter-workflow differences were no longer observed after expert adjustment (WebCeph+). However, the Bland–Altman findings indicated that residual individual-level variability remained for selected parameters. These findings underscore that AI-assisted cephalometry is not yet suitable for unsupervised clinical use [13,27,51]. Clinically, the present findings suggest that although fully automated systems offer clear time advantages, their use without an expert verification step may lead to deviations that exceed pragmatic clinical thresholds and are likely to influence diagnosis and treatment planning. In practice, AI-based cephalometric outputs should therefore be interpreted as decision-support tools that require specialist oversight, rather than as standalone replacements for manual or semi-automated analyses [41,51].

Because vendor algorithms and software builds are periodically updated, the reliability and agreement of AI-assisted cephalometric tools should be re-evaluated over time. In this context, the present four-arm design may serve as a useful framework for periodic re-validation studies.

In interpreting the present findings, manual tracing was considered the conventional clinician-dependent reference workflow for agreement analyses. As an independent absolute reference standard was not available, the results primarily characterize agreement among the evaluated workflows rather than absolute measurement accuracy.

The strengths of this study include the simultaneous comparison of manual, semi-automated, and fully automated workflows under identical imaging and calibration conditions. Although the final primary analytic sample met the a priori calculated minimum sample size for the planned intermethod comparisons, its limited size may reduce the precision of parameter-specific estimates and restrict the generalizability of the findings. All human-dependent tracings were performed by a single examiner with 10 years of experience, which ensured procedural standardization and reduced inter-observer variation as a source of noise. However, although the use of a single experienced examiner improved standardization, it may also have limited the external generalizability of the findings. Landmark identification remains operator dependent, and the observed agreement between human-dependent and AI-assisted workflows may differ when assessments are performed by clinicians with different levels of experience. Therefore, the present findings should be interpreted as reflecting a standardized single-examiner setting rather than a multi-operator clinical environment. The absence of an inter-observer reliability assessment thus represents a methodological limitation.

A further limitation is that intramethod repeatability was evaluated in a relatively small randomly selected subset of eight radiographs based on the initial statistical planning approach. Although this assessment provided supportive information regarding within-workflow repeatability, the precision and generalizability of the ICC estimates may be limited by the size of the repeated-measurement subset. Therefore, the repeatability findings should be interpreted cautiously.

An additional limitation concerns the WebCeph+ adjustment workflow. Although each tracing workflow was completed separately rather than in parallel, and landmarks were corrected only when they were judged to be clearly misplaced on visual inspection of the source image, the investigator performing the corrections was not formally blinded to the outputs of the other methods. Therefore, confirmation bias cannot be fully excluded. Prior familiarity with manual and Dolphin tracings may have influenced the correction process to some extent.

Additional limitations include the single-center design, the absence of subgroup analyses according to malocclusion type, and unresolved data-security considerations related to web-based platforms. Although the radiograph-level exclusion of cases containing statistically extreme values was planned a priori and applied to preserve within-subject consistency across method comparisons, the possibility that this procedure excluded more challenging cases and thereby limited the generalizability of the findings cannot be entirely excluded. Future studies should incorporate multiple examiners to quantify inter-observer agreement, apply blinded correction protocols with multiple independent experts, and evaluate AI performance under non-ideal imaging conditions such as poor superimposition, asymmetry, and motion artefacts, which more closely reflect routine clinical variability.

In summary, fully automated cephalometric analysis was not fully interchangeable with manual or semi-automated methods across all parameters in the present study. However, expert-assisted adjustment improved the agreement of AI-generated measurements with clinician-dependent workflows in the present dataset, although residual individual-level variability remained for selected parameters, suggesting that AI may currently be more appropriately used as a clinician-supervised supportive tool rather than as a stand-alone substitute.

## 5. Conclusions

Fully automated WebCeph analysis was not fully interchangeable with manual tracing or semi-automated Dolphin analysis for several clinically relevant skeletal and soft-tissue measurements. However, expert adjustment of AI-generated landmarks substantially improved agreement and reduced systematic mean discrepancies, although residual individual-level variability remained for selected parameters. Therefore, AI-based cephalometric analysis may currently be best considered a supportive clinical tool that requires expert verification rather than an unsupervised replacement for conventional methods.

## Figures and Tables

**Table 1:** Cephalometric indicators selected for skeletal, dental, and soft-tissue analysis.

Skeletal Parameters	Dental Parameters	Soft Tissue
SNA	U1 to SN (degrees)	Nasolabial angle
SNB	U1 to FH (degrees)	Mentolabial angle
ANB	IMPA	
FMA	Interincisal angle	
Y-Axis angle	Overjet	
Gonial angle	Overbite	
Posterior angle sum (Jarabak)		
Co-Gn		
Go-Gn		
A point to N-perpendicular		
Pogonion to N-perpendicular		
S-N		
Wits appraisal		

**Table 2:** Patient demographic data.

	Female (*n* = 35)	Male (*n* = 19)	Total (*n* = 54)	*p* Value *
Age	15.2 ± 2.00	14.68 ± 2.33	15.0 ± 2.13	0.428 ^a^
(Mean ± SD)				
Sex	35 (64.8%)	19 (35.2%)	54 (100%)	**0.03 ^b^**
(*n* %)				

* *p* value < 0.05 was considered statistically significant; bold values indicate statistically significant differences. ^a^ Mann–Whitney U Test; ^b^ Chi-square test.

**Table 3:** Intraclass correlation coefficients (ICCs) of repeated cephalometric measurements obtained by manual tracing, Dolphin, WebCeph, and WebCeph+ for assessment of intramethod repeatability.

Measurement	Intraclass Correlation Coefficient (ICC)			
Parameters	Manual Tracing	Dolphin	WebCeph	WebCeph+
SNA	0.920	0.968	1.00	0.905
SNB	0.972	0.988	1.00	0.932
ANB	0.915	0.957	1.00	0.965
FMA	0.935	0.907	1.00	0.982
U1-SN	0.988	0.962	1.00	0.962
U1-FH	0.987	0.967	1.00	0.952
IMPA	0.972	0.978	1.00	0.947
Y-Axis angle	0.925	0.977	1.00	0.942
Interincisal angle	0.972	0.988	1.00	0.963
Nasolabial angle	0.981	0.926	1.00	0.998
Gonial angle	0.906	0.975	1.00	0.994
Mentolabial angle	0.975	0.958	1.00	0.999
Posterior angle sum (Jarabak)	0.912	0.998	1.00	0.971
Co-Gn	0.978	0.959	1.00	0.987
Go-Gn	0.942	0.989	1.00	0.990
A to N-perpendicular	0.983	0.903	1.00	0.990
Pog to N-perpendicular	0.988	0.871	1.00	0.983
S-N	0.947	0.909	1.00	0.923
Wits	0.970	0.992	1.00	0.995
Overjet	0.983	0.973	1.00	0.979
Overbite	0.994	0.984	1.00	0.994

**Table 4:** Mean and standard deviation of cephalometric parameters obtained using Manual Tracing, Dolphin, WebCeph, and WebCeph+, along with the corresponding ANOVA results for inter-method comparisons.

Measurement	Manual Tracing	Dolphin	WebCeph	WebCeph+	*p* * Value
Parameters	(Mean ± SD)	(Mean ± SD)	(Mean ± SD)	(Mean ± SD)	
Measurements in angular					
SNA	(81.3 ± 3.86) ^ab^	(80.1 ± 3.92) ^b^	(82.3 ± 3.58) ^a^	(81.3 ± 3.9) ^ab^	**0.03**
SNB	(77.2 ± 3.8) ^a^	(75.8 ± 3.81) ^a^	(77.3 ± 3.39) ^a^	(76.8 ± 3.75) ^a^	0.143
ANB	(4.1 ± 2.16) ^a^	(4.3 ± 2.11) ^a^	(5.0 ± 1.96) ^a^	(4.4 ± 1.85) ^a^	0.107
FMA	(22.5 ± 4.48) ^a^	(22.5 ± 4.39) ^a^	(24.5 ± 4.69) ^a^	(22.6 ± 4.74) ^a^	0.066
Y-Axis angle	(57.8 ± 3.37) ^a^	(57.1 ± 3.62) ^a^	(59.5 ± 2.84) ^a^	(56.8 ± 3.18) ^a^	0.076
Gonial angle	(122.1 ± 5.39) ^a^	(122.3 ± 5.71) ^a^	(123.3 ± 5.68) ^a^	(122.7 ± 5.57) ^a^	0.681
Posterior angle sum (Jarabak)	(393.7 ± 5.2) ^a^	(394.4 ± 4.99) ^a^	(393.4 ± 5.07) ^a^	(393.3 ± 5.07) ^a^	0.657
Interincisal angle	(122.8 ± 12.12) ^a^	(128.3 ± 11.16) ^a^	(126.3 ± 9.99) ^a^	(124.9 ± 9.94) ^a^	0.063
U1 to SN	(104.3 ± 7.94) ^a^	(101.9 ± 7.34) ^a^	(103.2 ± 6.84) ^a^	(104.5 ± 6.61) ^a^	0.228
U1 to FH	(115.5 ± 7.88) ^a^	(113.9 ± 6.82) ^a^	(112.3 ± 6.37) ^a^	(115.5 ± 6.52) ^a^	0.058
IMPA	(96.4 ± 7.18) ^ab^	(95.2 ± 6.99) ^a^	(99.1 ± 6.49) ^b^	(96.8 ± 6.14) ^ab^	**0.027**
Nasolabial angle	(105.1 ± 9.97) ^a^	(104.7 ± 7.56) ^a^	(110.7 ± 10.46) ^b^	(103.0 ± 6.34) ^a^	**<0.001**
Mentolabial angle	(120.9 ± 7.3) ^a^	(122.6 ± 6.5) ^a^	(129.5 ± 13.58) ^b^	(121.5 ± 7.1) ^a^	**0.006**
Measurements in mm					
Co-Gn	(104.8 ± 4.7) ^a^	(104.7 ± 4.55) ^a^	(104.6 ± 4.47) ^a^	(104.9 ± 4.49) ^a^	0.98
Go-Gn	(69.1 ± 2.99) ^a^	(69.4 ± 3.39) ^a^	(66.3 ± 2.25) ^b^	(68.7 ± 2.59) ^a^	**0.018**
A point to N-perpendicular	(1.9 ± 3.36) ^a^	(2.0 ± 3.05) ^a^	(1.4 ± 2.69) ^a^	(2.2 ± 3.06) ^a^	0.582
Pogonion to N-perpendicular	(−2.5 ± 5.86) ^a^	(−2.3 ± 5.55) ^a^	(−4.8 ± 4.63) ^b^	(−2.4 ± 5.35) ^a^	**0.032**
S-N	(64.7 ± 2.98) ^a^	(65.6 ± 2.43) ^a^	(65.0 ± 2.33) ^a^	(65.5 ± 2.43) ^a^	0.203
Wits appraisal	(0.9 ± 1.38) ^a^	(1.1 ± 1.32) ^a^	(3.4 ± 1.7) ^b^	(1.3 ± 1.35) ^a^	**<0.001**
Overjet	(4.2 ± 2.12) ^a^	(4.1 ± 2.03) ^a^	(4.3 ± 1.99) ^a^	(4.1 ± 2.15) ^a^	0.934
Overbite	(2.2 ± 1.99) ^a^	(2.0 ± 2.05) ^a^	(1.8 ± 2.02) ^a^	(2.3 ± 1.86) ^a^	0.608

* One-Way ANOVA. ^a,b^: Means sharing at least one common superscript letter within the same row are not significantly different from each other in post hoc pairwise comparisons, whereas means with no common superscript letters differ significantly at *p* < 0.05. Bold values indicate statistically significant differences.

## Data Availability

Raw data supporting the findings of this study are available from the corresponding author on request.

## Abbreviations

The following abbreviations are used in this manuscript: AI, Artificial Intelligence; DICOM, Digital Imaging and Communications in Medicine; ICC, Intraclass Correlation Coefficient; SPSS, Statistical Package for Social Sciences; PACS, Picture Archiving and Communication System.
